# New Contributions to Asarum Powder on Immunology Related Toxicity Effects in Lung

**DOI:** 10.1155/2018/1054032

**Published:** 2018-09-02

**Authors:** Yamin Li, Lintao Han, Chunhua Huang, Wangqiang Dai, Guangyu Tian, Fang Huang, Jingjing Li, Jinwei Liu, Qiong Wang, Zhenxiang Zhou

**Affiliations:** College of Basic Medicine, Hubei University of Chinese Medicine, Wuhan, China

## Abstract

*Objective. *Asarum is widely used in clinical practice of Chinese medicine in the treatment of respiratory diseases. Many toxic ingredients (safrole, etc.) had been found in Asarum that show multiple visceral toxicities. In this study, we performed systematic investigation of expression profiles of genes to take a new insight into unclear mechanism of Asarum toxicities in lung.* Methods.* mRNAs were extracted from lungs of rats after intragastric administration with/without Asarum powders, and microarray assays were applied to investigate gene expression profiles. Differentially expressed genes with significance were selected to carry out GO analysis. Subsequently, quantitative PCRs were performed to verify the differential expression of Tmprss6, Prkag3, Nptx2, Antxr11, Klk11, Rag2, Olr77, Cd7, Il20, LOC69, C6, Ccl20, LOC68, and Cd163 in lung. Changes of Ampk, Bcl2, Caspase 3, Il1, Il20, Matriptase2, Nf*κ*b, Nptx2, and Rag2 in the lung on protein level were verified by western blotting and immunohistochemistry.* Results. *Compared with control group, the estimated organ coefficients were relatively increased in Asarum group. Results of GO analysis showed that a group of immune related genes in lung were expressed abnormally. The result of PCRs showed that Ccl20 was downregulated rather than other upregulated genes in the Asarum group. Western blotting and immunohistochemistry images showed that Asarum can upregulate the expression of Ampk, Caspase 3, Il1, Il20, Matriptase2, Nf*κ*b, and Rag2 and downregulate the expression of Bcl2 in lung.* Conclusion. *Our data suggest that expressions of immune related genes in lung were selectively altered by Asarum. Therefore, inflammatory response was active, by regulating Caspase 3, Il1, Il20, Matriptase2, Nf*κ*b, Rag2, Tmprss6, Prkag3, Nptx2, Antxr1, Klk11, Olr77, Cd7, LOC69, C6, LOC68, Cd163, Ampk, Bcl2, and Ccl20. Our study indicated that inflammatory factors take effect in lung toxicity caused by Asarum, which provides a new insight into molecular mechanism of Asarum toxicities in lung.

## 1. Introduction

Asarum is a controversial traditional Chinese medicine [1, 2] with anti-inflammatory, analgesic, antiarrhythmic, and anticancer activities [3–6]. Asarum was broadly used in compound prescriptions to treat respiratory diseases in traditional Chinese medicine. It was found that Asarum showed experimental effect of antibronchospasm [7]. Asarum also showed expectorant effect after compatibility [8]. Ginger moxibustion and acupoint sticking therapy by Asarum and white mustard can effectively improve the clinical symptoms of bronchial asthma [9].

Asarum is a genus of plant in the birthwort family Aristolochiaceae [10]. Various toxins were found in different parts of Asarum plant [11], including major toxins such as aristolochic acids [12, 13], safrole [14, 15], terpinolene [4, 16, 17], methyleugenol [15], sarisan [18, 19], 3,5-dimethoxy, Nutmeg [20] and other minor toxins including lignans [21], ketone [22], vinyl [16], benzene derivatives [23], Methyleugenol [24], and phenanthrene derivatives [25].

Remarkably, Asarum contains Aristolochene [25, 26], which exists broadly in Aristolochia plants, and showed extremely strong oncogenic [27], mutagenic, [28] and renal toxicity [13, 29, 30]. Sarisan can lead to carcinogenesis through liver metabolism [19] and respiratory disorders by inhibiting nerve transfer in medullary the neural networks [31].

Recently, a number of reports have investigated the molecular mechanisms of immune regulating activities of Asarum. Zhang et al. found that Asarum extract may plays an anti-inflammatory role by regulating the NF*κ*B and MAPK signaling pathways to treat induced arthritis [3]. Ku, S.K, and other investigators found that the active substances of Asarum pellitorine and episesamin can be used as a candidate drug for the treatment of severe vasculitis diseases. Pellitorine can inhibit TNF-*α*, IL-6, NF*κ*b, and extra cellular regulated kinase (ERK) 1/2 and then inhibit the HMGB1 signaling pathway to treat a variety of serious vasculitic disease. Episesamin can inhibit the expression of TNF-*α*, IL-1*β*, and TACE, can reduce P38 phosphorylation, ERK1/2, and c-Jun amino terminal kinase(JNK), which was induced by Phorbol-12-myristate 13-acetate(PMA) stimulation, and then can treat inflammatory disease of serious vascular [32, 33]. It was also found that the chemical constituents of Asarum roots can reduce the expression of COX-2 and iNOS genes, which provide a scientific basis for the development of new drugs to treat inflammatory diseases and metabolic diseases by using Asarum as the raw material [34].

However, molecular mechanism of immune regulation activity of Asarum was poorly understood, this undetermined situation overshadows the safety and accuracy of clinical medication of this broadly used herb. In this study, we investigated and analyzed the expression patterns of immune related genes, and this work may help reveal the molecular mechanism of Asarum toxicity in lung.

## 2. Materials and Methods

### 2.1. Reagents and Antibodies

Reagents and antibodies included the following: Trizol (bioteke China); YBR Green PCR kit (Takara China); reverse transcription kit (Takara China); qPCR reaction system (Tsingke Biological Technology); BeyoECL Star (Beyotime Biotechnology); ani-caspase-3 antibody (Rabbit abcam); anti-Bcl-2 antibody (Rabbit abcam); anti-IL-1*β* antibody (Rabbit abcam); anti-Ampk gamma 3 antibody (Rabbit abcam); anti-IL-20 receptor alpha antibody (Rabbit abcam); anti-RAG2 antibody(Rabbit abcam); anti-NPTX2 antibody(Rabbit abcam); anti-Matriptase 2 antibody (Rabbit abcam); NF*κ*Bp65(Rabbit CST); *β*-Actin(i102) polyclonal antibody (Rabbit Bioworld); secondary antibody (Goat anti-Rabbit abcam); protein maker (Thermo).

### 2.2. Animal Research

40 SPF grade male Sprauge-Dawley (SD) rats were purchased from the experimental animal research center of Hubei Province, (license number: ZCXK, Hubei 2015-0018). The rats were given ordinary diet and drinking at room temperature between 18-25°C and relative humidity about 60-70%, with natural circadian rhythm and light. The 40 rats were randomly divided into two groups: 20 were in the control group and 20 were in the Asarum group. Intragastric administrating began after one week of adaptive feeding with dried powder of Asarum root (model group) or saline (blank group). For 28 days of intragastric administration was performed with a dosage of 1.35g Asarum/kg•day. Rats were killed 4 weeks later by giving 4% chloral hydrate as anesthetic to alleviate the pain of rats. Blood was taken from abdominal aortic, and then each organ is weighted. 1 cm*∗*1cm samples of lung tissues were put into 4% paraformaldehyde and then embedded in paraffin and cut into sections of 4 millimeters thick for the next immunohistochemical staining. 100 mg of lung tissue samples was homogenated in 1 ml Trizol to extract RNA for consequential microarray detection and quantitative PCRs assays, or in 1 ml RIPA for Western blotting. 100 mg samples of lung tissues were also kept in liquid nitrogen. The experiment plan and nursing were carried out under the supervision of animal ethics committee of Hubei University of traditional Chinese medicine.

### 2.3. Microarray

Rat lung genes expressions were profiled by Agilent Rat Gene Expression template (8*∗*60K Design ID:028279) chip, cRNA and chip in hybrid hybrid 16-20h and the chip box, washing and staining in the washing station, and then scanning. Using single color fluorescence labeling method, each group was detected by an individual chip. By comparing the signals of two chips, it was possible to explore the differential expressions of genes. Feature Extraction software (version10.7.1.1, Agilent Technologies) was used to process the original image to extract the original data. Subsequently, Genespring software (version 12.5; Agilent Technologies) was used for processing quantile standardization. The notable value of differential gene expression analysis was screened by t-test, with p values<0.05 and fold changes more than 2. Gene ontology consortium (http://www.geneontology.org/) was used to perform differential expression gene ontology (GO) enrichment analysis. Pathway analysis was used to determine the significant pathway of the differential genes on the basis of Kyoto Encyclopedia of Genes and Genomes (KEGG) (http://www.genome.jp/kegg/pathway.html).

### 2.4. RNA Extraction and Real Time Reverse Transcription Polymerase Chain Reaction (RT-PCR)

Total lung tissue RNA was extracted by Trizol reagent according to the instructions of the kit. Reverse transcription was performed by cDNA using reverse transcription system (Takara, Japan). 1 *μ*l cDNA, 1 *μ*l primers, and SYBR Green PCR Master Mix mixed (Takara, Japan) with the final volume of 25 ul. The PCR reaction conditions included 43 cycles of 95°Cfor 15 sec, 50°Cfor 30 sec, and 72°C for 20 sec, and the dissolution curves were measured at 50-95°C, with an increase rate of 0.5°C for 5 sec at the CFX96 quantitative PCR system (Biorad, USA). The threshold cycle (CT) value was determined by quantitative PCRs, and the housekeeping gene beta actin was normalized. 2^−ΔΔCT^ method was used to calculate the relative gene expression levels. The RT-PCR primers are shown in Table 1.

### 2.5. Western Blotting Analysis

Protein concentrations of homogenated samples were determined by Coomassie brilliant blue method.12%SDS-polyacrylamide gel electrophoresis was used to separate proteins. Protein bands were transferred on PVDF membranes by 100V electrophoresis for 70 minutes. PVDF membranes were sealed with 5% skimmed milk at room temperature, 2 hours for antigen and antibody binding reaction of anti-caspase-3 antibody (1:100), and anti-Bcl-2 antibody (1:500); anti-IL-1*β* antibody (1:5000); anti-Ampk antibody (1: 100); anti-IL-20 receptor *α* antibody (1:100); anti-RAG2 antibody(1:500); anti-NPTX2 antibody(1:1000); anti-matriptase 2 antibody (1:1000); NF*κ*Bp65 (1:1000). After 2 hours, at room temperature, washing was done 6 times*∗*5min with TBST containing 0.5% Tween 20. After cleaning, secondary antibody was used with horseradish peroxide to react. After 1 h hours at room temperature, washing was done 6 times*∗*5min with TBST containing 0.5% Tween 20. The ChemiDoc imaging system (Biorad, USA) was used to image and analysis PVDF membranes, to determine relative expressions of the target proteins.

### 2.6. Immunohistochemistry

The lung tissue samples were embed and fixed and then immunohistochemical stained with following antibodies: anti-caspase3 antibody; anti-Bcl2 antibody; anti-Il1 beta antibody; anti-Ampk gamma antibody anti-Il20 receptor alpha 3; antibody; anti-Rag2 antibody; anti-Nptx2 antibody; anti-matriptase 2 antibody; Nf*κ*bp65. After washing, samples were labeled by secondary antibodies and revealed color with DAB. The differences between the Asarum group and the control group are recorded by microscopical photographing.

## 3. Results

### 3.1. Asarum Effects Visceral Organ Coefficients in Rats

In the third weeks after intragastric administration, the rats became listlessness, the periphery of the eyes and auricles appeared cyanosis, and hair appeared messy and gloss. At the end of the intragastric administration, the organs were weighed and the organ coefficients were calculated. In model group, the results of organ coefficients, liver function, and renal function were significantly higher than control group (Table 2).

### 3.2. Asarum Effects Gene Expression Patterns in Lung

**Figure 1:**
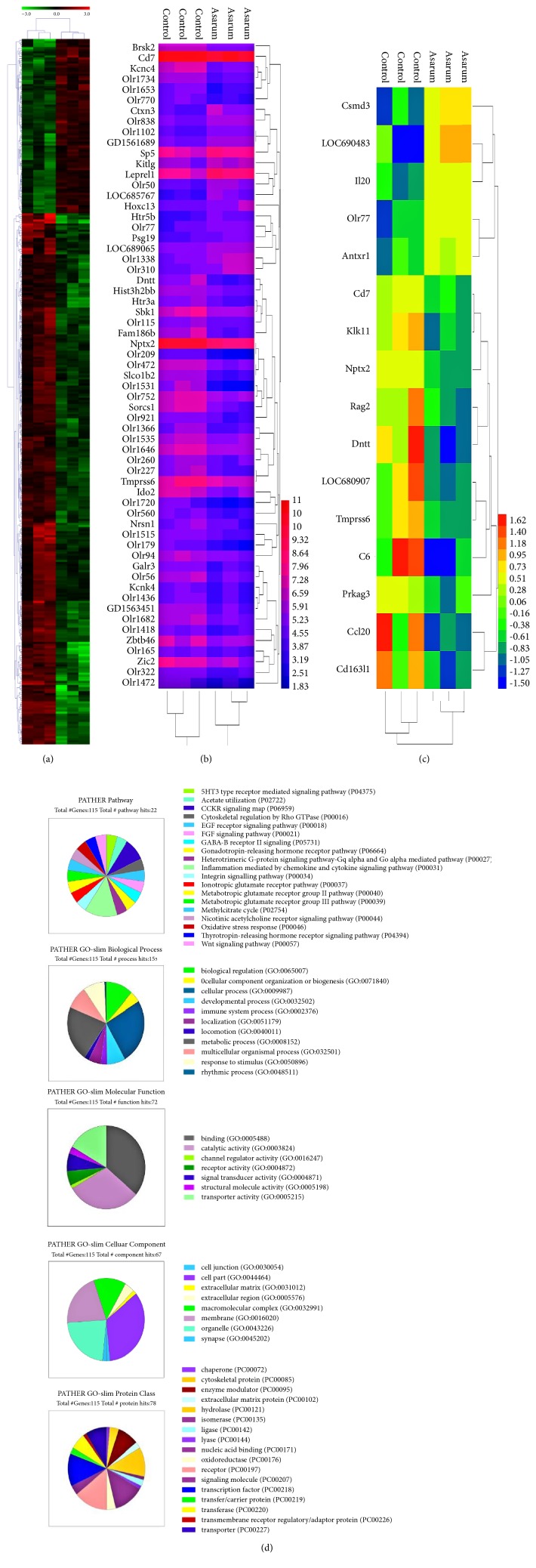
The effect of Asarum on the genes level changes in lung tissues of rats. Gene chip technology was performed to detect the genes expression of lungs in rats after intragastric administration with/without Asarum powders. The results showed that, after treating with Asarum, 344 genes changed obviously in the lung tissues, 259 genes significantly upregulated, and the other 85 genes significantly decreased. The results also showed that the immune related genes express varies. The results may be induced by the toxicity of Asarum on lung in rats.

The results showed that, after treating with Asarum, 344 genes changed obviously in the lung tissues, 259 genes significantly upregulated, and the other 85 genes significantly decreased (Figure 1(a)). The intersection analysis of all differential expressed genes, by pathway, was made with the biological pathways of common databases KEGG, BioCart, Transpath, and GenMAPP. The pathway analysis of differentially expressed genes in the KEGG database shows that metabolism is mainly concentrated in metabolic pathways. Signal transduction pathways involving most genes are in the olfactory transduction pathway, MAPK signaling pathways, and calcium signaling pathways. And signaling molecules interacting with the most genes are involved in neuroactive ligands. The cell process is mainly transport and catabolism in receptor interaction. The organismal system mainly is focused on immune system. The involving diseases mainly are cancer, immune system disease, kidney transsexual disease, and so on (Table 3). According to the results, it is shown that the lung cells in Asarum group involving various GPCR based signaling pathways were maybe related to the upregulation of olfactory receptor and immune system. The results also showed that the immune related genes express varies. So we removed the olfactory related factors and analyzed the remaining genes in gene ontology consortium. The results showed that changes came in the expression of immune related genes (Figure 1(b)). Then, we put immune related genes in the gene ontology consortium to GO analysis (Figure 1(c)). The PATHER Pathway, PATHER GO-Slim Biological Process, PATHER GO-Slim Molecular Process, PATHER GO-Slim Cellular Component, and PATHER Protein Class were all applied to analyze the genes (Figure 1(d)). The results of PATHER Pathway showed that inflammation related genes played a key role. The results of PATHER GO-Slim biological process showed that cellular process and metabolic process are the most percentage. The results of PATHER GO-Slim Molecular Process showed that binding and catalytic activity are the most. The results of PATHER GO-Slim Cellular Component showed that cell part and membrane are the most. The results of PATHER Protein Class showed that receptor and transcription factor are the most. We chose the immune related genes such as the upregulated genes Tmprss6, Prkag3, Antxr1, Klk11, Rag2, Olr77, Cd7, Il20, LOC69, C6, LOC68, and Cd163 and downregulated genes Ccl20 to verify the results of microarrays with the method of quantitative PCRs.

### 3.3. Expressions Patterns of Inflammatory Factors Were Altered by Asarum

**Figure 2:**
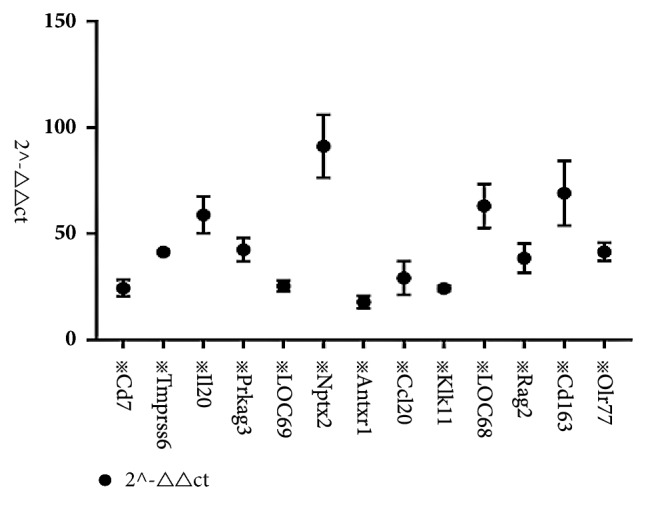
The effect of Asarun on the RNA level changes in lung tissues of rats. qRT-PCR was performed to detect the mRNA expression of Tmprss6(matriptase 2), Prkag3, Nptx2, Antxr1, Klk11, Rag2, Olr77, Cd7, Il20, LOC69, C6, Ccl20, LOC68, and Cd163 in lungs of rats after intragastric administration with/without Asarum powders. The results showed that Ccl20 was downregulated rather than other upregulated genes in the Asarum group, compared with the control group after intragastric administration with Asarum powders. That may be induced by the toxicity of Asarum. The data were the 2^−∆∆ct^ of at least three independent experiments. All comparisons were performed with WPS office (kingsoft, China), utilizing unpaired t-test with Welch's correction. Statistical significance was marked as***※*** while p<0.05. *※*represent the significant difference between the Asarum group and control group (p<0.05).

**Figure 3:**
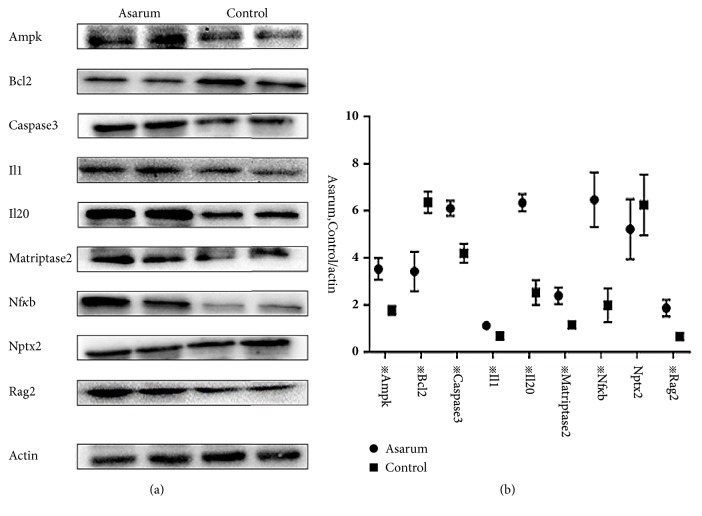
The effect of Asarum on the protein level changes in lung tissues of rats. Western blotting was performed to detect the protein expression of Ampk, Bcl2, Caspase 3, Il1, Il20, Matriptase2, Nf*κ*bp65, Nptx2, and Rag2 in lungs of rats after intragastric administration with/without Asarum powders. The results showed that the expressions of the Ampk, Caspase 3, Il1, Il20, Matriptase2, Nf*κ*bp65, and Rag2 were upregulated after intragastric administration with Asarum powders, the expression of Bcl2 was downregulated. The expression of Nptx2 was meaningless. The results may be induced by the toxicity of Asarum on lung in rats. The data were the means ± standard error of at least three independent experiments. All comparisons were performed with WPS office (kingsoft, China), utilizing unpaired t-test with Welch's correction. Statistical significance was marked as***※*** while p<0.05. *※*represent the significant difference between the Asarum group and control group (p<0.05).

**Figure 4:**
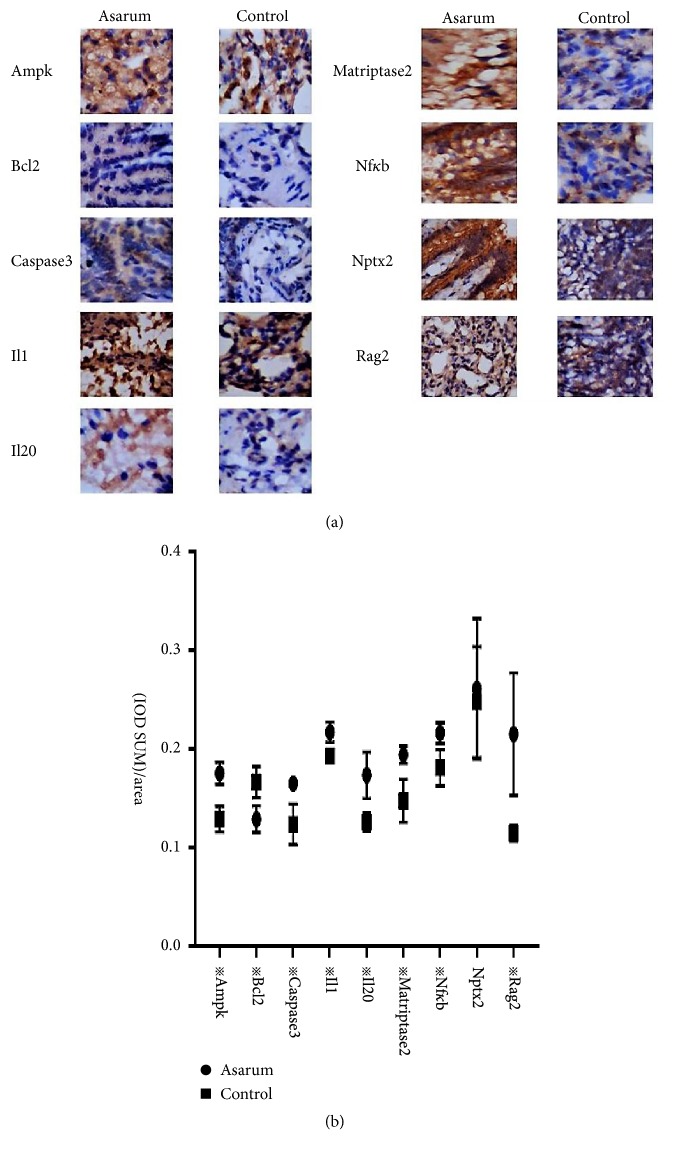
The effect of Asarun on morphology changes in lung tissues of rats. Immunohistochemistry was performed to detect the expression of Ampk, Bcl2, Caspase 3, Il1, Il20, Matriptase2, Nf*κ*bp65, Nptx2, and Rag2 in lungs of rats after intragastric administration with/without Asarum powders. The results showed that the expressions of the Ampk, Caspase 3, Il1, Il20, Matriptase2, Nf*κ*bp65, and Rag2 were upregulated after intragastric administration with Asarum powders and the expression of Bcl2 was downregulated. The expression of Nptx2 was meaningless. The results may be induced by the toxicity of Asarum on lung in rats. The data were the means ± standard error of at least three independent experiments. All comparisons were performed with WPS office (kingsoft, China), utilizing unpaired t-test with Welch's correction. Statistical significance was marked as***※*** while p<0.05. *※*represent the significant difference between the Asarum group and control group (p<0.05).

The results of quantitative PCRs showed that the expression levels Tmprss6, Prkag3, Antxr1, Klk11, Rag2, Olr77, Cd7, Il20, LOC69, C6, LOC68, and Cd163 were upregulated in Asarum group relative to control group, but the expression of Ccl20 was more downregulated in Asarum group than in control group (Figure 2). Western blotting data also showed that the expressions of immune related genes including Ampk, Caspase 3, Il1*β*, Il20, Matriptase2, Nf*κ*b, and Rag2 were significantly upregulated; otherwise, the expressions of Bcl2 were significantly downregulated. The expression of Nptx2 was meaningless. (Figure 3). In addition, immunohistochemical test showed that the expressions of Ampk, Bcl2, Caspase 3, Il1*β*, Il20, Matriptase2, Nf*κ*bp65, and Rag2 of each group were different with statistically significant P < 0.05 (Figure 4).

## 4. Discussion

On the toxicity of Asarum, the views of medical practitioners from the past dynasties to present are inconformity. It is Chen Cheng in Song Dynasty who firstly mentioned Asarum toxicity and its dose limit. The Chinese Pharmacopoeia stipulates that the dosage of Asarum is 1-3g in decoction without referring to toxicity. But, it is widespread to use Asarum with overdose in clinical. So, it is of great significance to study the toxic mechanism of Asarum to the body. In this study, we focused on the toxicity mechanism of Asarum on lung.

In this study, the rats in the model group were treated with Asarum powder and the toxic reaction was observed. The toxicities were based on the detection of organ coefficients, liver, and kidney function. We detected the differences between the two groups. Based on the results we determine whether the model was successful. Organ coefficient is a common indicator of chronic toxicity [35, 36]. We clarified that Asarum can lead to increased organ coefficients. Compared with the control group, the results of liver and kidney function were remarkably higher. In summary, the results indicated that our model was success and long-term intake of Asarum powder could lead to obvious lung toxicity. This lays a solid foundation for the next step of our experiment.

Microarray [37] is a multigene expression profile scanning method based on the principle of hybridization sequence sequencing, relatively fast and efficient. It is widely used in the diagnosis of diseases, drug researches, and new drugs discovery. The effect of traditional Chinese medicine on the human body is multicomponent, multitarget, and multiroute [38]. Microarray technology is characterized by high sensitivity and high throughput, which provides a basis for the study of Asarum lung toxicity. Therefore, the analysis of different genes in each group can be used to study the regulation mechanism of Asarum toxicity on lung more systematically. However, few studies have focused on the gene expression of Asarum lung toxicity, and the relationship between gene expressions has not been confirmed. The results of this study showed that there were significant differences in gene expression between two groups. The altered genes are related to the olfactory expression receptor and the expression of inflammation related genes, which are related to the activation of the immune factors. Olfactory receptor expression changes may be associated with the nature (pungent and warm) and meridian distribution (lung) of Asarum. The production of Asarum lung toxicity may be caused by changes in the expression of the olfactory receptor to produce the immune response, which leads to the activation of the inflammatory response.

According to our results, it may be inferred that genes changes of Asarum lung occur before the target organ damage. The pulmonary toxicity of Asarum may be an important cause and important segment in the damage of the lung and other target organs. The damage of lung function is closely related to the level of inflammation and oxidative stress [39]. In our study, microarray results showed that Asarum lung toxicity is associated with the differential expression of inflammatory related factors. Differentially expressed genes with significance were selected to carry out GO analysis. Subsequently, quantitative PCRs were performed to verify the differential expression of Tmprss6, Olr77, Rag2, Il20, Cd7, Cd163, LOC69, LOC68, C6, Ccl20, Prkag3, Antar1, Klk11, and Nptx2 in lung. The results showed that, except for Nptx2, Ccl20 was downregulated rather than other upregulated genes in Asarum group, compared with blank group.

Type II transmembrane serine protease (Tmprss6) participates in a variety of physiological and pathological processes, such as cell cycle, cell proliferation and migration, and cell apoptosis. It has reported that Tmprss6 may alert olfactory neurons to produce iron starvation [40]. Olfactory receptor (Olr77) is a G protein coupled receptor. Anthrax toxin receptor 1 (Antxr1), also known as capillary morphogenesis gene 1 (CMG1), is a transmembrane receptor protein and ligand binding of anthrax toxin and can activate its downstream signaling pathway and mediate its toxic part into the cell play effect. The human tissue kallikrein family (human kallikrein KLK) is a subfamily of serine protease, is a serine protease activity substance which predicted that Klk11 may be involved in the cascade reaction, catalytic extracellular barrier damage. Overexpression of Klk11 can promote the occurrence of lung inflammation in rats. The expressions of Tmprss6, Olr77, Klk11, and Antxr1 were higher in the model group than the blank group. The results showed that the first link of the pungent herb-Asarum acts on body is likely to start in the olfactory receptor. The activation of the olfactory receptors causes a series of downstream reaction, resulting in a “pungent” effect. It may has an enlightening effect on the study of multitarget and multipathway of medicinal properties of traditional Chinese medicine.

The function of the immune system, to a large extent, depends on interleukin [41]. The immune response of the body is closely related to the function of B and T cells. B cells, T cells, complement, and chemokines regulate the immune response of the body together. B cells can be differentiated into plasma cells under antigen stimulation. Plasma cells can synthesize and secrete antibodies (immunoglobulin), which mainly perform humoral immunity. A variety of cytokines regulate the activation, proliferation, and differentiation of B cells by binding to the cytokine receptors corresponding to the surface of B cells. The cytokine receptors of B cells are mainly interleukins. A variety of cytokines can act on T cells, which is due to the expression of cytokines on the surface of T cells, such as the interleukin receptor. The surface of B cells and T cells has the same cytokine interleukins. Chemokines also contain interleukin. In our study, we found the related genes (Rag2, Il20, Cd7, Cd163, and C6) got changed. Rag2 is expressed in mature mouse B cells by interleukin 4 and costimuli (lipopolysaccharide and other cytokines). Rag2 also can reconstruct a highly diverse immunoglobulin and T cell receptor through a combination of encoded DNA fragments [42, 43]. Il20 is a member of the interleukin 10 family, which can limit the damage caused by virus and bacterial infection and also help to heal infection or inflammation of the tissue damage process. Il10 family cytokines have an indispensable function in many infectious and inflammatory diseases [44]. Recently, studies have confirmed that Il20 plays an important role in various immune and inflammatory diseases such as bronchial asthma [45]. Cd7 belongs to the members of immunoglobulin superfamily. It is often expressed in T cells and natural killer cells and plays an important role in the interaction between T cells or T cells interacting with B cells [46]. Cd163 is a kind of protein of the scavenger receptor, which is expressed by phagocytic cells. It participates in many immune activities and plays an important role in normal physiology [47, 48]. Chemokine has been deemed to be the driver of leukocyte migration in the immune system. As an early inflammatory mediator, the interaction between chemokines, pathogenic microorganisms, and inflammatory cells determines the clinical manifestations and outcomes of many infectious diseases [49]. Chemokines can be divided into four family CXC, CC, C, and CX3C [50]. Ccl20 is a chemokine of CC subfamily discovered in recent years. It directly participates in the directional migration of dendritic cells and T cells through the action of ligand CCR6 and plays a role in immune and immune diseases [51]. Ccl20 also has strong broad-spectrum antibacterial activity [52]. Complement is one of the important immune system, which not only participates in the body's defensive effect and self-stability but also can cause immune injury. The activation products of complement release inflammatory regulators and promote tissue damage in the inflammatory sites and are related to the pathogenesis of some immune diseases [53]. In addition, complement can also enhance the body's humoral immunity to T cell dependent antigens and affect the critical level of B cell activation, thereby linking innate immunity and adaptive immunity [54]. The determination of C6 content is helpful for the diagnosis and treatment of various autoimmune diseases [55]. The results showed that the expressions of these genes were upregulated except that Ccl20 was downregulated, and we speculated that the production of Asarum lung toxicity can produce an inflammatory response through the role of interleukin factors, complement, and chemokines.

From the results, we can predict that the production of Asarum lung toxicity is closely related to the factors of inflammatory and immune related complements, chemokines, interleukins, olfactory receptors, Ampk, and so on. In this study, we found that Asarum has a significant effect on lung and significantly affected the gene expression patterns. We validated the differential genes by quantitative PCRs. The results of quantitative PCRs are in agreement with the results of gene. The results showed that, in protein level, we choose western blotting and immunohistochemistry to detect immune related proteins in order to see the changes. We found that Asarum can upregulate the expression of Ampk, Caspase 3, Il1*β*, Il20, Matriptase2, Nf*κ*bp65, and Rag2 and downregulate the expression of Bcl2 but not affect expression of Nptx2 significantly.

Prkag 3 is a constituent of Adenylate activated protein kinase (Ampk). Ampk is an important protein kinase, the main function of which is to coordinate metabolism and energy balance [56]. Ampk also is involved in the control of inflammation [57]. Studies had shown that Ampk mainly through the downstream proteins indirectly or directly regulates the inhibition of Nf*κ*b activity, then inhibiting the expression of inflammatory factors [58]. Nuclear factor kappa B (Nf*κ*b) is a transcription factor that regulates the expression of multiple genes. It has been proved to regulate multiple cellular functions, including inflammation and stress response and survival [59]. Ampk-Nf*κ*b pathway plays an important and significant regulatory role in lung inflammatory response. The organism can downregulate the activity of transcription factors and the expression of inflammatory related genes through Ampk-Nf*κ*b to reduce inflammatory damage. Many inflammatory substances were regulated by Nf*κ*b, such as Il1*β* and Il20. It was also found that metformin protects against acute lung injury caused by LPS, and the function may be related to the activation of Ampk [60]. Studies also showed that the expression levels of Il1, Nf*κ*b, and Caspase3 were significantly increased in lung injury induced by blast, while the expression level of Bcl2 was decreased [61]. Bcl2 inhibits apoptosis, inflammation, proliferation, and differentiation [62]. Il1 is composed of mononuclear cells, endothelial cells, fibroblasts, and other cell types in response to infection of cytokines produced by Caspase, which participates in growth, differentiation, and apoptosis regulation of cell. Caspase3 is a cysteine protease, which is a key enzyme that causes cell apoptosis. Once activated by signal pathway, it can degrade proteins in cells and make cells irreversible to death [63]. Nptx2 is a novel proinflammatory cytokine that can predict AD correlation, which is better than any other immunological marker, and is mainly related to brain atrophy, especially memory decline [64]. Although Nptx2 is one of the factors that are closely related to the inflammatory response, there is no difference between the Asarum group and the control group.

These results suggested that Asarum may induce lung toxicity by altering the balance of energy metabolism in body, such as bleeding, edema, and other inflammatory changes. The results had guiding significance for the selection of Asarum dosage and the molecular mechanism of toxicological pharmacology. Therefore, the toxic mechanism of Asarum to the lung may be through the immune response produced by inflammatory factors, thereby changing the expression of these genes. According to the above results, we know that Asarum may though affect the Ampk-Nf*κ*b pathway, Bcl2 pathway, and inflammation associated proteins to lead to inflammatory reaction to produce lung toxicity, but the mechanism needs further investigation. Overall, this study showed that Asarum has pulmonary toxicity, which can cause the organ coefficients to change and can lead to inflammation related genes change. The results of western blotting, quantitative PCRs, and gene expression are identical. Therefore, this may be new understanding of the toxic effects of Asarum on lung, but more studies need to be done to clarify the mechanism of lung toxicity with Asarum.

## Figures and Tables

**Table 1:** RT-PCR primers.

Target gene	Forward/Reverse	Sequence(5'-3')
Prkag3	Forward	CTCTGGCACTGTGCTCTACA
	Reverse	GCCCACGACTTGACCAGATT
Cd7	Forward	CGAGGCCGTCAGAAAAGTCA
	Reverse	ACACAGTTTCTTGATCTGCGTC
Rag2	Forward	GCCTTCAGTGCCAAAATAAGAG
	Reverse	CTCTTAGGCCAGCCTTTTTGG
Nptx2	Forward	TCAACGACAAGGTCGCACAG
	Reverse	ACTGGCTAAGCTCTCCAACG
LOC690483	Forward	GAGCTCAGAAAGACCTGACCC
	Reverse	TCTGAGAGTGGTGTCCTGGT
Ccl20	Forward	CACCTCCTCAGCCTAAGAACC
	Reverse	GCCCCTCATAGATTGTGGGA
Il20	Forward	CAGTCTGTCATTCTCACATGGC
	Reverse	CTTGGACAGGAGCGTTCTCA
Olr77	Forward	ATCTGGGGTGCTAGTGGCTA
	Reverse	TAACAGAACGGGTGGGAAAGG
LOC680907	Forward	ACCTGGCTGATGCAGAAGTT
	Reverse	CTGAGGAATTGCCTGATGCCA
Klk11	Forward	AGAAAGGCGGAAAGGGCCTA
	Reverse	TCCCCCTACGTGCCCTGTTA
Tmprss6	Forward	TCCACTATGTCCGATGGCTG
	Reverse	GCGGAACCATAGGGCTTTGA
Antxr1	Forward	GAGGGAGGCTAACAGATCCC
	Reverse	GGTGGAAGGTTCAGCTGCTA
Cd163l1	Forward	TCTCTGTGCAAATGGCACCT
	Reverse	TCTGCAGTTACGGATGGTGG
C6	Forward	GTGTGTGTGCCAGAGTGGTA
	Reverse	TGCACGGTTGTCCAACTTTT
beta-actin	Forward	CGCGAGTACAACCTTCTTGC
	Reverse	ATACCCACCATCACACCCTGG

**Table 2:** Organ coefficients and functions.

Organ coefficients and functions	blank	Asarum
organ coefficients(g/100g), n=7		
lung	0.40±0.05	0.56±0.07*∗*
liver	3.65±0.39	4.23±0.30*∗*
heart	0.31±0.01	0.40±0.03*∗*
kidney	0.53±0.07	0.71±0.03*∗*

liver function, n=5		
TBil	2.66±0.39	29.2±3.65*∗*
ALT	46.2±2.71	64±5.58*∗*
AST	40±9.87	77.2±16.83*∗*

renal function, n=5		
TP	72±4.81	55.8±2.4*∗*
ALB	46.4±4.67	36.8±0.75*∗*
GELO	22.2±1.72	17.8±1.72*∗*
BUN	2.36±0.26	7.28±0.78*∗*
CR	16.8±2.13	26.4±2.57*∗*

Note: *∗* represent Asarum compare with blank p<0.05. To analyze the toxicity of Asarum to rats after intragastric administration with/without Asarum powders with the detection of organ coefficient, liver and kidney function. The results showed that the above data were all up-regulated after intragastric administration with Asarum powders. That may be induced by the toxicity of Asarum. The data were the means ± standard error of at least three independent experiments. All comparisons were performed with WPS office (kingsoft, China), utilizing unpaired t-test with Welch's correction. Statistical significance was marked as *∗* while p<0.05. *∗*represent the significant difference between the Asarum group and control group (p<0.05).

**Table 3:** KEGG pathway.

Gene name	KO	Pathway class	system	Pathway name
Pipox	K00306	Metabolism	Global and overview maps	Metabolic pathways
Afmid	K01432	Metabolism	Global and overview maps	Metabolic pathways
Cyp4a1	K07425	Metabolism	Global and overview maps	Metabolic pathways
B4galnt4	K09657	Metabolism	Global and overview maps	Metabolic pathways
Afmid	K01432	Metabolism	Carbohydrate metabolism	Glyoxylate and dicarboxylate metabolism
Cyp4a1	K07425	Metabolism	Lipid metabolism	Fatty acid degradation
Cyp4a1	K07425	Metabolism	Lipid metabolism	Arachidonic acid metabolism
Gucy2d	K12321	Metabolism	Nucleotide metabolism	Purine metabolism
Pipox	K00306	Metabolism	Amino acid metabolism	Glycine, serine and threonine metabolism
Pipox	K00306	Metabolism	Amino acid metabolism	Lysine degradation
Afmid	K01432	Metabolism	Amino acid metabolism	Tryptophan metabolism
B4galnt4	K09657	Metabolism	Glycan biosynthesis and metabolism	Various types of N-glycan biosynthesis
Cyp4a1	K07425	Metabolism	Metabolism of cofactors and vitamins	Retinol metabolism
Kitlg	K05461	Environmental Information Processing	Signal transduction	Ras signaling pathway
Kitlg	K05461	Environmental Information Processing	Signal transduction	Rap1 signaling pathway
Kitlg	K05461	Environmental Information Processing	Signal transduction	MAPK signaling pathway
Kitlg	K05461	Environmental Information Processing	Signal transduction	Phospholipase D signaling pathway
Kitlg	K05461	Environmental Information Processing	Signal transduction	PI3K-Akt signaling pathway
Cacna1e	K04852	Environmental Information Processing	Signal transduction	MAPK signaling pathway
Cacna1e	K04852	Environmental Information Processing	Signal transduction	Calcium signaling pathway
Il20	K22667	Environmental Information Processing	Signal transduction	Jak-STAT signaling pathway
Rag2	K10988	Environmental Information Processing	Signal transduction	FoxO signaling pathway
Tacr1	K04222	Environmental Information Processing	Signal transduction	Calcium signaling pathway
Tacr1	K04222	Environmental Information Processing	Signaling molecules and interaction	Neuroactive ligand-receptor interaction
Galr3	K04232	Environmental Information Processing	Signaling molecules and interaction	Neuroactive ligand-receptor interaction
Il20	K22667	Environmental Information Processing	Signaling molecules and interaction	Cytokine-cytokine receptor interaction
Lrrc4	K16351	Environmental Information Processing	Signaling molecules and interaction	Cell adhesion molecules (CAMs)
Pipox	K00306	Cellular Processes	Transport and catabolism	Peroxisome
Kitlg	K05461	Organismal Systems	Immune system	Hematopoietic cell lineage
Cd7	K06457	Organismal Systems	Immune system	Hematopoietic cell lineage
C8b	K03998	Organismal Systems	Immune system	Complement and coagulation cascades
Cyp4a1	K07425	Organismal Systems	Endocrine system	PPAR signaling pathway
Kitlg	K05461	Organismal Systems	Endocrine system	Melanogenesis
Cyp4a1	K07425	Organismal Systems	Circulatory system	Vascular smooth muscle contraction
Fxyd4	K13359	Organismal Systems	Excretory system	Aldosterone-regulated sodium reabsorption
Kcnq3	K04928	Organismal Systems	Nervous system	Cholinergic synapse
Cplx3	K15295	Organismal Systems	Nervous system	Synaptic vesicle cycle
Gucy2d	K12321	Organismal Systems	Sensory system	04744 Phototransduction
Gucy2d	K12321	Organismal Systems	Sensory system	Olfactory transduction
Cyp4a1	K07425	Organismal Systems	Sensory system	Inflammatory mediator regulation of TRP channels
Lrrc4	K16351	Organismal Systems	Development	Axon guidance
Kitlg	K05461	Human Diseases	Cancers: Overview	Pathways in cancer
C8b	K03998	Human Diseases	Immune diseases	Systemic lupus erythematosus
Rag2	K10988	Human Diseases	Immune diseases	Primary immunodeficiency
C8b	K03998	Human Diseases	Neurodegenerative diseases	Prion diseases
Cacna1e	K04852	Human Diseases	Endocrine and metabolic diseases	Type II diabetes mellitus
Pax4	K08032	Human Diseases	Endocrine and metabolic diseases	Maturity onset diabetes of the young
Tacr1	K04222	Human Diseases	Infectious diseases: Viral	Measles
C8b	K03998	Human Diseases	Infectious diseases: Parasitic	Amoebiasis

## Data Availability

The data used to support the findings of this study are available from the corresponding author upon request.
